# Parosmia as a predictor of a better olfactory function in COVID-19: a multicentric longitudinal study for upper respiratory tract infections

**DOI:** 10.1007/s00405-022-07781-1

**Published:** 2022-12-22

**Authors:** Susanne Menzel, Antje Haehner, Dorothea Woosch, Belinda Marquardt, Cristina Ressel, Julia Draf, Giancarlo Ottaviano, Paolo Boscolo-Rizzo, Romina Kardashi, Katja de With, Yvonne Hackl, Thomas Hummel

**Affiliations:** 1 Smell and Taste Clinic, Department of Otorhinolaryngology, University Hospital Carl Gustav Carus, Technische Universität Dresden, TU Dresden Faculty of Medicine Carl Gustav Carus: Technische Universitat Dresden Medizinische Fakultat Carl Gustav Carus, Fetscherstrasse 74, 01307 Dresden, Germany; 2 Department of Neurosciences-ENT Section, University-Hospital of Padova, Padua, Italy; 3 Department of Medicine, Surgery and Health Sciences, Section of Otolaryngology, University of Trieste, Trieste, Italy; 4 Division of Infectious Diseases, University Hospital Carl Gustav Carus, Technische Universität Dresden, Dresden, Germany; 5 Department of Otorhinolaryngology and Head and Neck Surgery, University Hospital Augsburg, Sauerbruchstraße 6, 86179 Augsburg, Germany

**Keywords:** Smell, Olfactory dysfunction, COVID-19, Parosmia

## Abstract

**Purpose:**

This study aimed to evaluate the course of olfactory dysfunction [OD] due to upper respiratory tract infections [URTI] especially for COVID-19 [C19] in a multicentric design and to investigate possible predictors for the outcome.

**Methods:**

In a multicentric study, patients (*n* = 147, of which 96 were women) with OD due to URTI, including C19 and non-C19 were evaluated at two visits with a standardized medical history and “Sniffin’ Sticks” extended psychophysical testing to examine the course and possible predictors for improvement of olfactory function.

**Results:**

C19 patients showed better overall olfactory function (*p* < 0.001) compared to non-C19. Olfactory function (*p* < 0.001) improved over 3.5 ± 1.2 months in a comparable fashion for C19 and non-C19 comparable over time (*p* = 0.20) except for a more pronounced improvement of odour threshold (*p* = 0.03) in C19. C19 patients with parosmia exhibited a higher probability of clinically relevant improvement of odour threshold, a better threshold in the second visit, and tended to have a better TDI-score at the second visit. Further possible predictors for an improving olfactory function were younger age, female gender, and had lower scores in olfactory tests at the first visit.

**Conclusions:**

Patients with C19 and non-C19 URTI exhibit a similar improvement over 3–4 months except for the odour threshold, with a better TDI in both visits for C19. For C19 a better prognosis in terms of olfactory recovery was found for younger patients with parosmia and lower olfactory scores at the first visit. Still, for many patients with olfactory loss, an improvement that is experienced as complete may only occur over months and possibly years.

## Introduction

Olfactory dysfunction (OD) can be categorized into quantitative and qualitative impairment. Quantitative OD means a change of the sensitivity to olfactory stimuli, such as hyper-, hypo- or anosmia. While hyperosmia is rarely observed [1], the prevalence of hypo- and anosmia is about 19% [2] to 25% [3]. Apart from aging the most common causes of acquired quantitative OD are sinonasal diseases (prevalence 30–74% of all smell disorders), upper respiratory tract infection (URTI 11–25%), and traumatic brain injury (TBI 5–14%) [4, 5]. Due to the ongoing C19 pandemic, the cases of associated OD are dramatically increasing. Loss of smell and/or taste is reported in up to 88% [6] (48% [7]; 34% [8]; 15% [9]) of these patients depending on the variant of the virus [10].

Qualitative OD can be divided into parosmia, which is defined as distorted odour perception in the presence of an odour, and phantosmia, which is an odour perception without an odour source [11]. Qualitative OD is most commonly present along with smell loss due to—in descending frequency URTI, TBI, idiopathic OD, or sinonasal causes [12, 13]. Qualitative OD in C19 and other causes is associated with poorer quality of life [14, 15]. Interestingly, some studies showed an association of parosmia with better olfactory recovery [16, 17]. However, others found little prognostic value of parosmia [18] or no correlation between parosmia and olfactory recovery [19].

Olfactory dysfunction (OD) can have a tremendous impact on daily life, including problems to detect harmful substances, reduced food enjoyment, insecurity in social situations, or depression [20, 21]. Given the consequences for the patient, information about the course of the disorder is essential for patients and health professionals who are involved in their treatment.

For URTI, the probability of an improvement in olfactory function has been reported to be 32–36% during an observation period of 14 months with an average duration of disease of 17 or 16 months, respectively [22, 23]. For C19-associated OD as part of URTI, there is still limited data on the course of the disease. The majority of patients improve within the first 2 months in subjective ratings and objective testing of olfactory function [9]. Some studies showed a significant improvement in olfactory function within the first 6 months, of those 95% had an improvement with psychophysical tests [24], respectively, 88% with self-ratings [25]. Interestingly the majority of patients rating their olfactory function as normal after having C19 tend to have lasting olfactory loss [26]. Persistent OD has been shown in 27% 1 year after the COVID infection [27].

This study aimed to evaluate the course of OD due to URTI especially for C19-associated OD in a multicentric design and to investigate possible predictors for the outcome.

## Methods

### Participants

In a prospective study design, patients who presented themselves with OD due to URTI were recruited from outpatient clinic consultations of the following cities: Dresden (Germany) (*n* = 116; 80.3%), Augsburg (Germany) (*n* = 22; 15.0%) and Trieste (Italy) (*n* = 7; 4.8%). A total of 147 patients were included (age 18–83 years; mean age 44.3 ± 15.5 years). Depending on the cause of URTI the patients were divided into groups: C19-associated OD (*n* = 110, 74.8%) and non-C19 (*n* = 37, 25.2%), see Table 1. Patients with C19-related OD had been reported to be diagnosed via a reverse-transcription–polymerase chain reaction (RT–PCR) or antigen rapid test. Patients with a history of sudden olfactory loss and a negative reverse-transcription–polymerase chain reaction (RT–PCR) or antigen rapid test were categorized as non-C19-related OD.

**Table 1:** Descriptive data of the participants

Parameter	All OD						COVID-19						Non-COVID-19					
	Number	Percentage	Mean	SD	Min	Max	Number	Percentage	Mean	SD	Min	Max	Number	Percentage	Mean	SD	Min	Max
Participants	147	100%					110	74.8%					37	25.2%				
Gender																		
Women	96	65.3%					69	62.7%					27	73.0%				
Men	51	34.7%					41	37.3%					10	27.0%				
Age (in years)	146		44.3	15.5	18	83	109		41.8	14.2	18	78	37		51.8	16.9	20	83
Olfactory function																		
Threshold (1)	147		3.6	2.9	0	12.5	110		4.0	3.0	0	12.5	37		2.4	2.1	1	7.5
Discrimination (1)	147		9.9	3.0	0	16	110		10.2	2.8	0	16	37		8.9	3.6	0	14
Identification (1)	147		9.3	3.5	0	16	110		9.5	3.4	1	16	37		8.5	3.5	0	16
TDI-Score (1)	147		22.7	7.7	4	38.5	110		23.7	7.4	4	38.5	37		19.9	7.8	6	33.3
Threshold (2)	146		5.4	3.5	0	13.8	109		6.0	3.4	0	13.8	37		3.5	3.1	1	13
Discrimination (2)	146		11.6	2.7	0	16	109		12.0	2.4	4	16	37		10.4	3.2	0	15
Identification (2)	146		10.5	3.0	2	16	109		10.8	2.9	3	16	37		9.6	3.0	2	15
TDI-Score (2)	146		27.5	7.3	3	42	109		28.8	6.7	8	42	37		23.5	7.7	3	38
Parosmia (min. once)	72	49.3%					64	58.2%					8	21.6%				

SD standard deviation, Min minimum, Max maximum, (1) first visit, (2) second visit

The study was conducted according to the declaration of Helsinki and had been approved by the local ethics boards. All participants gave written informed consent.

### Procedure

The course of the OD was documented in two visits, which included the same procedure: a detailed medical history was taken including age, gender, duration of symptoms, and questions regarding parosmia and phantosmia. Patients underwent olfactory tests, using Sniffin’ Sticks test battery (Burghart Messtechnik, Wedel, Germany) to categorize the olfactory function: a score of fewer than 16 points indicates functional anosmia, between 16 and 31 points indicates hyposmia, and 31 points or more indicates normosmia [28, 29]. Furthermore, retronasal testing (using different powders in a short or extended test version) [30] and trigeminal testing (using the AmmoLa^®^ stick or acetic acid) were done [31]. Gustatory tests were mainly done using four taste sprays or in an extended version with “taste strips” [32, 33]. The classification of parosmia was based on the temporal occurrence (daily), intensity, and further consequences (e.g., weight loss).

### Statistical analysis

Patient records were assigned to codes and anonymized. For data processing, Microsoft Excel Office 365 version 2017 database (Microsoft Corp., Redmond, WA, USA) was used. Statistical analyses were performed using IBM SPSS Statistics (Statistical Packages for the Social Sciences, version 21.0, SPSS Inc., Chicago, Illinois, USA). Descriptive statistics were obtained; continuous variables are expressed as means with standard deviation, while categorical variables are presented as frequencies (percentages).

The group of C19 and non-C19-associated OD were compared using *t* tests for independent samples to assess differences in metric variables like age, and duration of disease for each visit separately. A chi-square test was conducted to compare the above-mentioned groups regarding nominal or ordinal scaled variables like gender. To compare the occurrence of parosmia between C19 and non-C19 the Fisher test was used. Correlation analyses were performed according to Pearson. Mann–Whitney *U* test was used for the comparison of the categorization of olfactory function within the visits. To evaluate a clinically relevant olfactory change, we divided the patients regarding the difference in threshold, discrimination, and identification, resp. TDI-score between the first and the second visit into two groups: ≥ 2.5 resp. ≥ 5.5 (for TDI) with clinically relevant change, < 2.5 resp. < 5.5 (TDI) without. A *p* value of < 0.05 was considered statistically significant.

## Results

### Study population

Slightly more women (96, 65.3%) than men were included in the study. However, the groups of C19 and non-C19 did not differ concerning gender (*χ*^2^(1) = 1.28, *p *= 0.26, *ɸ *= − 0.09).

Patients with C19 patients were younger (mean= 41.8 ± 14.2 years) than non-C19 patients (mean = 51.8 ± 16.9 years; *t*(144) = 3.56; *p* < 0.001; 95% CI[− 15.68, − 4.49]).

The duration of the disease was on average 7.7 ± 5.4 months. Patients with C19 had a shorter duration of disease than non-C19 patients (mean = 6.2 ± 3.3 months vs. 12.1 ± 7.8 months; *t*(40.47) = − 4.45; *p* < 0.001; 95% CI [− 8.53, − 3.21]).

Patients returned for a second visit after 3.4 ± 1.0 months (mean ± SD). There was a difference between C19 and non-C19 (*t*(136.4) = 2.54; *p* = 0.012; 95% CI [0.07, 0.59]), with a shorter interval of the second visit for non-C19 (3.2 ± 0.5 months) compared to patients with C19 (3.5 ± 1.1 months). The difference of 10 days appeared to be without clinical importance.

### Comparison of C19 vs. non-C19-related OD

**Fig. 1:**
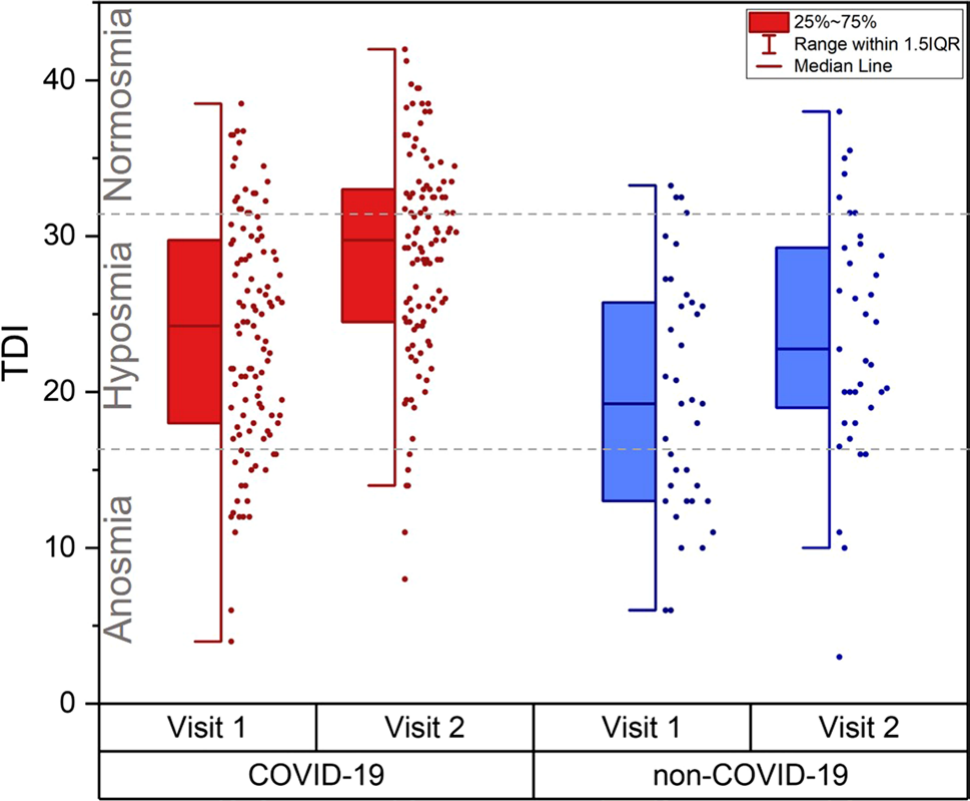
Comparison of TDI-scores first vs. second visit regarding the C19 status for URTI: for both, C19 and non-C19 an improvement from the first to the second visit was observed, even though C19 had better TDI-scores in both visits

C19 patients had a better olfactory function in the first and the second visit: patients who had C19 had a better odour threshold (*t*(91.80) = 3.50, *p* < 0.001, mean difference 1.56, 95% CI [0.67, 2.45]) and TDI-score in the first visit (*t*(145) = 2.69, *p* = 0.008, mean difference 3.84, 95% CI [1.02, 6.67]) (Fig. 1). Furthermore, C19 patients outperform non-C19 in the second visit for odour threshold (*t*(144) = 3.90, *p* < 0.001, mean difference 2.48, 95% CI [1.22, 3.73]), discrimination (*t*(144) = 3.23, *p* = 0.002, mean difference 1.60, 95% CI [0.62, 2.58]), identification (*t*(144) = 2.16, *p* = 0.032; mean difference 1.21, 95% CI [0.11, 2.32]) and TDI-score (*t*(144) = 3.99, *p* < 0.001, mean difference 5.29, 95% CI [2.67, 7.91]), see Table 1 and Fig. 1. For the discrimination (*p* = 0.06) and the identification (*p* = 0.12) conducted in the first visit was no difference found between C19 and non-C19. When comparing patients in age-matched groups in terms of the C19 status, younger (≤ 55 years, n = 88 with C19, *n* = 19 non-C19) patients with C19 had a better threshold in the first visit (*t*(46.68) = 2.65, *p* = 0.011, mean = 4.1 ± 3.1 vs. mean = 2.6 ± 2.2), a better threshold in the second visit (*t*(121) = 3.07, *p* = 0.003, mean = 6.1 ± 3.4 vs. 3.8 ± 3.3), and a better TDI-score in the second visit (*t*(121) = 2.36, *p* = 0.020, mean = 29.0 ± 6.8 vs. mean = 25.3 ± 7.2) than non-C19 patients. Older patients (> 55 years, *n* = 21 with C19, *n* = 18 with non-C19) with C19 had a better odour discrimination in the second visit (*t*(37) = 2.65, *p* = 0.012) and tended to have a better TDI-score in the second visit (*t*(37) = 1.81, *p* = 0.078) than non-C19.

The better olfactory function in C19 is also seen in the difference in the categorization of olfactory dysfunction between these two groups (Mann–Whitney *U* Test *U* = 1499.50, *Z* = − 2.54, *p* = 0.011 for the first visit; Mann–Whitney *U* Test *U* = 1524.50, *Z* = − 2.30, *p* = 0.022 for the second visit): in the first visit the majority of the C19 patients were hyposmic (*n* = 73, 66.4%), some were normosmic (*n* = 21, 19.1%) and a few were anosmic (*n* = 16, 14.5%). In the second visit, the majority was hyposmic (*n* = 58, 52.7%) with an increased number of normosmic (*n* = 45, 40.9%) and only a few were anosmic (*n* = 6, 5.5%). For non-C19 patients, the majority in the first visit were hyposmic (*n* = 26, 52.8%), with many being anosmic (*n* = 13, 36.1%) and only a few were normosmic (*n* = 4, 11.1%). In the second visit the vast majority were hyposmic (*n* = 26, 72.2%), a few were normosmic (*n* = 7, 19.4%) and anosmic (*n* = 3, 8.3%).

Although C19 patients had an overall better olfactory function, both non-C19 (TDI + 3.7 ± 4.7) and C19 (TDI + 4.9 ± 5.2) patients improved similarly (*p* = 0.20) comparing the TDI of first and the second visit, see Fig. 1. The ratio of an improved TDI between both visits was similar (*χ*^2^(1) = 1.16; *p* = 0.28) between C19 (85.5%, n = 94) and non-C19 (77.8%, *n* = 28), indicating that the majority of patients improved. Furthermore, both groups behaved similarly in terms of clinically relevant improvement of TDI: 43.1% (*n* = 47) for C19 and 30.5% (*n* = 11) for non-C19 (*χ*^2^(1) = 1.78, *p* = 0.18). When looking in detail at the individual parameters of olfactory function, non-C19 and C19 did not differ in the difference of odour discrimination (*p* = 0.51) or identification (*p* = 0.67) between both visits or clinically relevant improvement of odour discrimination (*p* = 0.51) nor identification (*p* = 0.63). However, patients with C19 showed a more pronounced improvement in odour threshold (2.1 ± 3.5; t(106.8) = 2.21, *p* = 0.03) than non-C19 patients (1.1 ± 2.1). This is also reflected in the more frequent clinically relevant improvement in odour threshold in C19 (41.3%, *χ*^2^(1) = 7.64, *p* = 0.006) patients than in non-C19 (16.2%).

Regarding the taste sprays a trend of slightly better results in C19 patients was shown in the first visit (*t*(80) = 1.73, *p* = 0.088, mean difference 0.48), but there was no difference between both groups in the second visit (*p* = 0.82). There were no differences between the two groups concerning the retronasal tests (*p* = 0.89 resp. *p* = 0.73) and trigeminal tests (*p* = 0.29 resp. *p* = 0.85) in either the first or second visit.

### Predictors for an improving olfactory function

#### Gender

A clinically relevant improvement of the TDI score was found more frequently in women than in men for all URTI (*χ*^2^(1) = 5.16, *p* = 0.023, 46.8% of women improved clinically relevant, 27.5% of men) and non-C19 (Fisher Test *p* = 0.015), but not for C19 (*p* = 0.14). Regarding the improvement of threshold, discrimination, identification, and TDI-score no difference in gender was found for all URTI (*p* = 0.16 to *p* = 0.44). C19 patients did not show any difference in gender for the improvement of threshold, discrimination, identification, or TDI-score (*p* = 0.13 to *p* = 0.77). For non-C19 patients, the TDI-score tended to improve more for women (*t*(34.7) = 1.85, *p* = 0.073, 4.3 ± 5.3) than for men (2.1 ± 1.7). There was no difference in gender for the improvement of threshold, discrimination, identification, or TDI-score (*p* = 0.16 to *p* = 0.96).

#### Age

Patients with URTI who had a clinically relevant improvement of their TDI and threshold, were younger than patients without such relevant improvement (mean = 40.4 ± 16.9 years vs. 46.8 ± 14.0 years, *t*(142) = 2.49, *p* = 0.014 resp. 38.9 ± 15.1 years vs. 47.0 ± 15.0 years, *t*(143) = 3.10, *p* = 0.002). Regarding a relevant improvement of discrimination (*p* = 0.12) or identification (*p* = 0.34) was no difference in age found for all URTI. C19 patients with a clinically relevant improvement of threshold were younger (*t*(107) = 3.16; *p* = 0.002, 36.8 ± 14.2 years vs. 45.1 ± 13.2 years) and tended to be younger (*t*(106) = 1.90, *p* = 0.061) in the group of clinically relevant TDI improvement (mean = 38.9 ± 15.6 years vs. mean = 44.1 ± 12.8 years). C19 patients with a clinically relevant improvement in discrimination were older (45.4 ± 15.8 years vs. 39.3 ± 12.5 years, *t*(107) = 2.27, *p* = 0.025). There was no difference in patients' age regarding a relevant improvement of identification (*p* = 0.50). For non-C19 patients, there was no difference in terms of age regarding a clinically relevant improvement of threshold (*p* = 0.70), discrimination (*p* = 0.93), identification (*p* = 0.82), and TDI (*p* = 0.344).

#### Olfactory function at the first visit

**Fig. 2:**
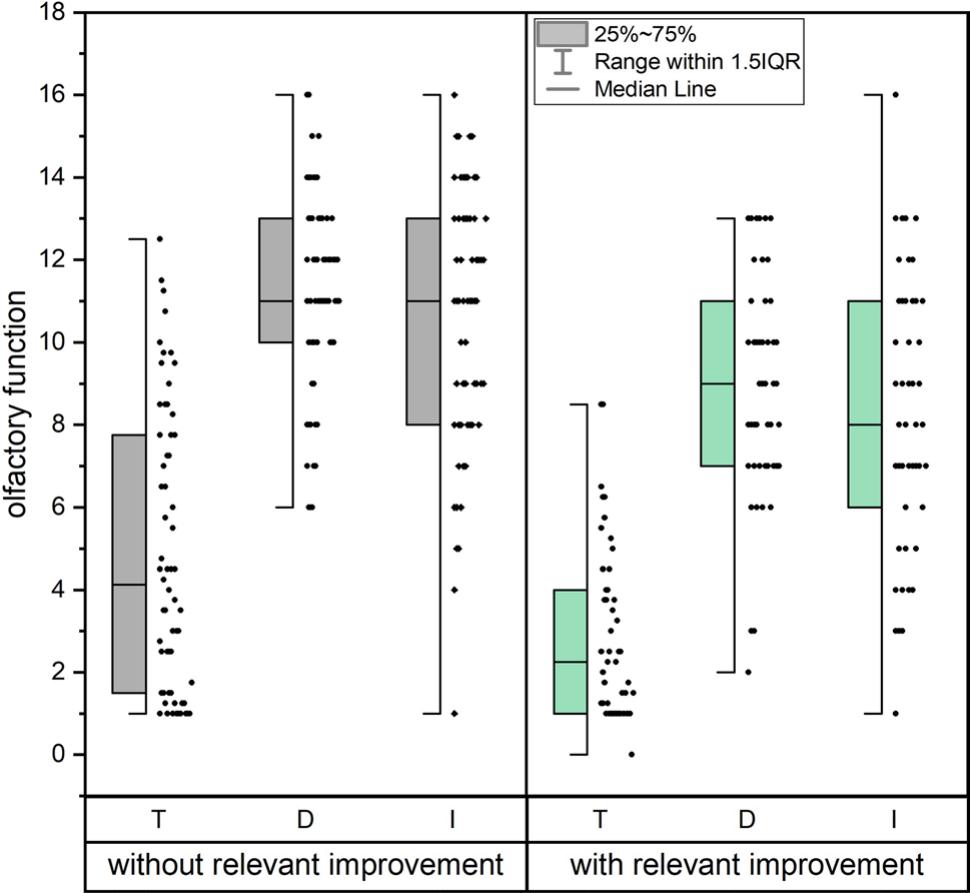
Box-Whisker-plot of odour threshold, discrimination, and identification for C19 patients as a function of clinically relevant improvement in olfactory function: C19 patients with a clinically relevant improvement of their olfactory function had initially a lower odour threshold, discrimination, and identification compared to those without a clinically relevant improvement

Patients with URTI in the group of clinically relevant improvement of TDI had initially in the first visit a lower threshold (*p* < 0.001), discrimination (*p* < 0.001), and identification (*p* < 0.001). C19 patients with a relevant improvement of their TDI-score had lower scores in the first visit for threshold (*p* < 0.001), discrimination (*p* < 0.001), and identification (*p* < 0.001), see Fig. 2. Non-C19 patients with a clinically relevant improvement of TDI showed a lower odour threshold (*t*(34.8) = 2.11, *p* = 0.042), identification (*t*(35) = 2.3, *p* = 0.024) and tended to have lower discrimination (t(35) = 1.70, *p* = 0.097).

#### Duration of disease

There was less improvement in TDI-score with a longer duration of OD (Pearson *r* = − 0.17, *p* = 0.046) for URTI, which is also seen in the trend of a slightly shorter duration of disease in the group of clinically relevant improvement (6.8 ± 4.4 months) compared to those without (8.2 ± 6.0 months, *t*(141.3) = 1.64, *p* = 0.10). For C19 we found no correlation (Pearson *r* = − 0.13, *p* = 0.19) of difference of TDI-score and the duration of disease nor a difference regarding the duration of disease in the groups with or without clinically relevant improvement (*p* = 0.23). For non-C19 patients, there was no difference in duration of disease regarding a clinically relevant improvement of TDI (*p* = 0.69) nor a correlation between TDI improvement and duration of disease (Pearson *r* = − 0.16, *p* = 0.35).

### Parosmia

**Fig. 3:**
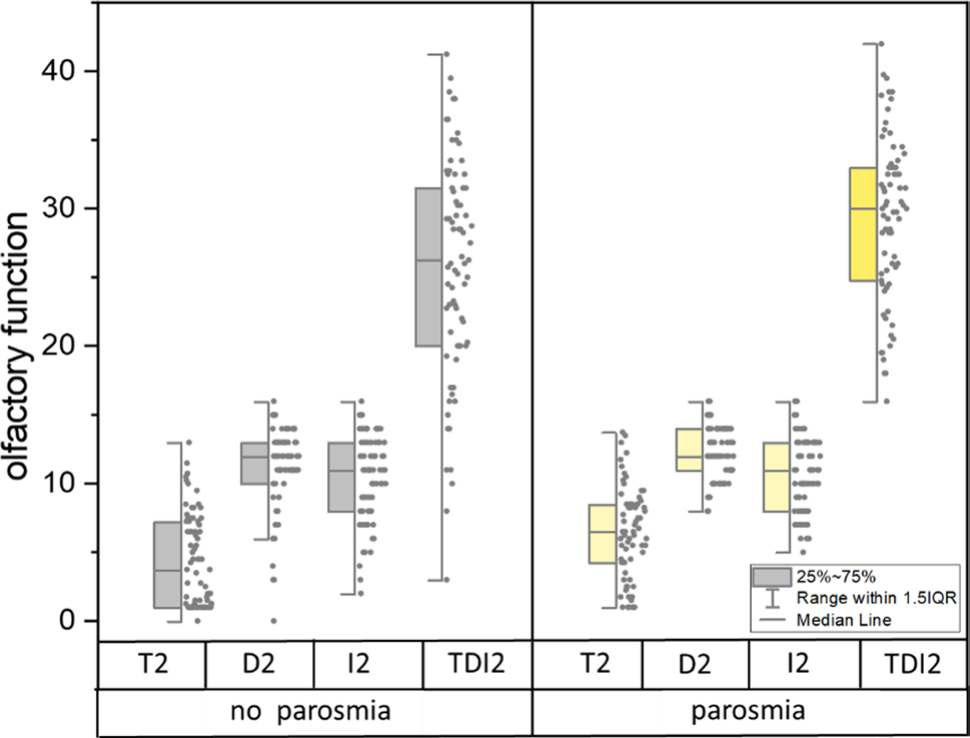
Box–Whisker-plot of odour threshold (T2), discrimination (D2), identification (I2), and TDI-scores (TDI2) of the second visit regarding an existing parosmia for URTI: in the second visit odour threshold, discrimination, and TDI-score of patients who had parosmia in one or both visits were better compared to patients who did not have parosmia

**Fig. 4:**
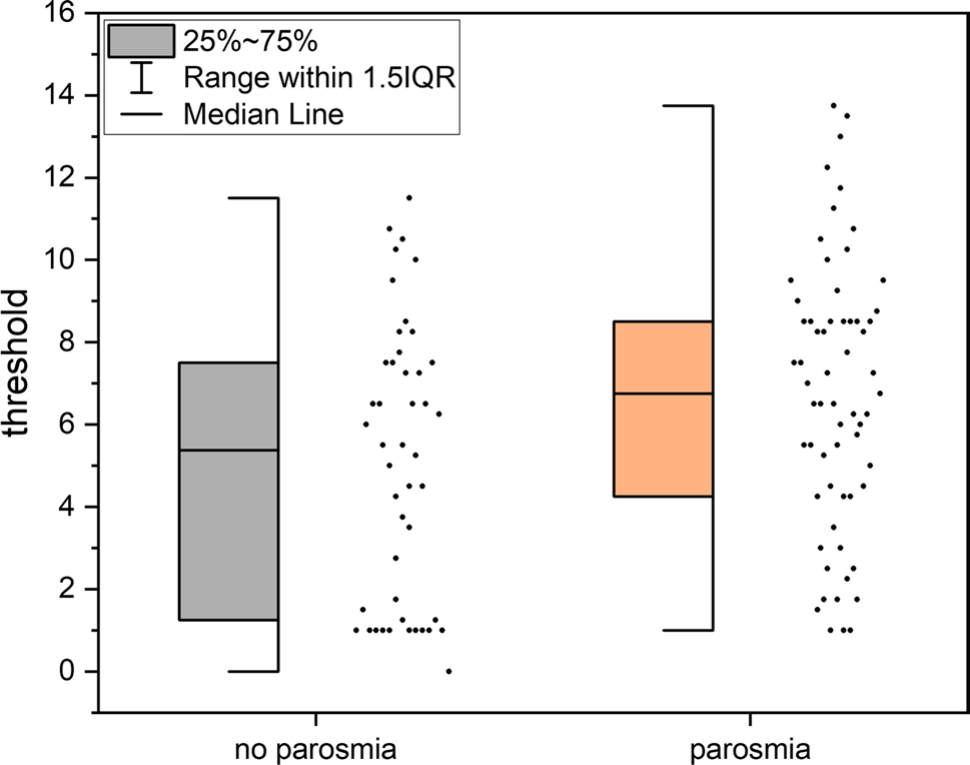
Box–Whisker-plot of odour threshold in the second visit for C19 patients regarding parosmia: C19 patients with parosmia for at least one visit had a better odour threshold in the second visit

Women had more frequently parosmia at one point than men [URTI: *χ*^2^(1) = 5.85, *p* = 0.016; 56.8% vs. 35.3%, C19: *χ*^2^(1) = 9.86, *p* = 0.002, but not for non-C19: Fisher test *p* = 1.00]. C19 patients had more frequent parosmia at least once in the two visits compared to non-C19 (*χ*^2^(1) = 14.81, *p* < 0.001, 58.2% vs. 21.6%), see Table 1. Patients with URTI who had parosmia at one visit had a better odour threshold (*t*(145) = 2.00, *p* = 0.048), discrimination (*t*(133.6) = 2.54, *p* = 0.013), and tended to have a better TDI-score (*t*(138.5) = 1.88, *p* = 0.062) in the first visit. Furthermore, URTI patients who had parosmia at one point had a better odour threshold (*t*(143) = 3.71, *p* < 0.001), odour discrimination (*t*(125.0) = 2.81, *p* = 0.006), and TDI-score (*t*(136.7) = 2.91, *p* = 0.004, 29.2 ± 6.0 vs. 25.9 ± 8.1) at the second visit (Fig. 3). There was no significant difference regarding parosmia at one point for the improvement of threshold (*p* = 0.11, 2.3 ± 3.0 vs. 1.5 ± 3.4), discrimination (*p* = 0.98), identification (*p* = 90), and TDI-score (*p* = 0.14, 5.3 ± 5.3 vs. 4.0 ± 5.0), although the improvement in TDI and threshold for parosmia borders on the clinically relevant range. C19 patients who had parosmia in one visit had better odour discrimination in the first visit (*t*(108) = 2.0, *p* = 0.048, 10.6 ± 2.4 vs. 9.6 ± 3.1), odour threshold in the second visit (*T*(107) = 2.65, *p* = 0.009, 6.7 ± 3.3 vs. 5.0 ± 3.3, see Fig. 4) and tended to have a better TDI-score in the second visit (*T*(107) = 1.83, *p* = 0.086, 29.8 ± 5.7 vs. 27.4 ± 7.8). There was no difference regarding the improvement of threshold (*p* = 0.32), discrimination (*p* = 0.66), identification (*p* = 0.99) or TDI-score (*p* = 0.24) between the two visits. For non-C19 patients there were no differences of threshold (*p* = 0.58 resp. 0.53), discrimination (*p* = 0.50 resp. 0.34), identification (*p* = 0.99 resp. 0.61), and TDI-score (*p* = 0.71 resp. 0.65) in the first or second visit regarding parosmia at one visit. In addition, no differences were found for the difference of threshold (*p* = 0.68), discrimination (*p* = 0.48), identification (*p* = 0.37), and TDI-score (*p* = 0.91) between both visits for non-C19.

**Fig. 5:**
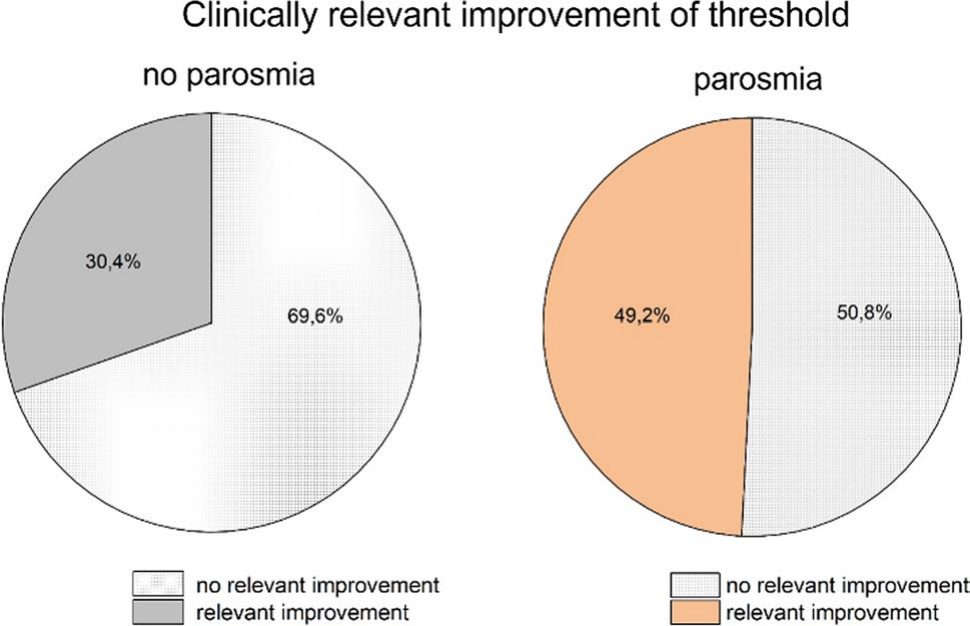
Pie chart of clinically relevant improvement of the threshold for existing parosmia for C19 patients: C19 patients who had parosmia in one visit had a higher proportion of clinically relevant improvement of their threshold

There is a higher proportion of those with a clinically relevant improvement in the threshold for existing parosmia in one visit for all URTI and C19 [URTI: *χ*^2^(1) = 8.11, *p* = 0.004, 46.5% with vs. 24.0% without parosmia; C19: *χ*^2^(1) = 3.87, *p* = 0.49, 49.2% with vs. 30.4% without, see Fig. 5], but not for non-C19 (*p* = 0.45). There were no differences between the occurrence of parosmia at one point and the proportion of clinically relevant improvement of odour discrimination (URTI *p* = 0.68; C19 *p* = 0.43, non-C19 Fisher test *p* = 1.00), identification (URTI *p* = 0.22; C19 *p* = 0.49, non-C19: Fisher test *p* = 0.079) or TDI-score (URTI *p* = 0.26, C19 *p* = 0.47, non-C19 Fisher test *p* = 1.00).

## Discussion

In this large multicentric study including patients with C19-associated OD and non-C19 URTI, the following main results emerged. First, patients with C19 had a better olfactory function than non-C19 patients. However, both groups improved similarly, except for odour threshold. Second, C19 patients had a better improvement of odour threshold than non-C19 patients. Third, C19 patients who reported parosmia in at least one visit had an increased threshold in the second visit, a higher proportion of clinically relevant improvement of odour threshold, and tended to have a better TDI-score in the second visit. Fourth, patients with a clinically relevant improvement of TDI had lower scores of olfactory function in the first visit, regardless of the C19 status. Furthermore, possible predictors for an improving TDI are younger age (for C19, but not for non-C19), female gender (for non-C19, but not for C19).

In our study, the TDI-score of patients with C19 and non-C19-related OD improved similarly except for odour threshold with a similar ratio of clinically relevant improvement of TDI. However, C19 patients showed a better overall olfactory function in both visits. In the age-matched comparison younger C19 patients outperformed younger non-C19 regarding odour threshold in both visits and TDI-score in the second visit. Older C19 patients had a better odour discrimination in the second visit and tended to have a better TDI-score in the second visit than age-matched non-C19 patients. In previous studies [34, 35] a worse olfactory function for C19 compared to non-C19 was shown for the acute course of the first weeks, which could indicate severe damage caused by SARS-CoV-2. An infection of SARS-CoV-2 can lead to temporal damage due to an inflammatory mechanism on sustentacular cells, stem cells, and perivascular cells, which could provoke an indirect impairment of olfactory receptor neurons. Furthermore, the damage of mainly non-neuronal cells at the level of the olfactory epithelium and vascular cells and a persistent downregulation of olfactory signalling genes within the olfactory receptor neurons could lead to a persistent olfactory dysfunction [36, 37]. In contrast, direct damage to olfactory sensory neurons is often described in non-C19-associated URTI, which can lead to a prolonged course [38–40]. Furthermore, it has been shown for non-C19 that the olfactory neuroepithelium is replaced by metaplastic squamous epithelium [41]. The pathophysiology of the severe damage at the peripheral level in non-C19 patients might explain the lower odour threshold in both visits for younger age-matched C19 patients, lower threshold improvement in the two visits and the lower proportion of a clinically relevant improvement of threshold. The recovery of olfactory function happens due to the regeneration of the olfactory mucosa starting from the basal cells [42, 43]. An initial very low olfactory function after a C19 infection appears to improve quite fast during the first month as previous studies showed [6, 9]. Nevertheless, there is a certain selection bias, as the groups of C19 and non-C19 differ in terms of age and in the duration of the disease. Nonetheless, in the age-matched analysis for the C19 patients, the better TDI score at the second visit persisted for all patients and a better threshold at both visits for the younger patients. If the duration of illness had been the same in both groups, a similar result might have been obtained. Currently, mainly patients with C19 present in the consultations for OD, which explains on one side the unbalanced ratio of C19 and non-C19 patients in our sample and on the second hand the longer duration of disease for the group of non-C19.

There are several theories for the occurrence of parosmia at the peripheral level of the olfactory epithelium as well as in central processing. A distorted perception of odours could be due to altered integration and interpretation of odours. On a peripheral level, a loss of receptor neurons reduces sensory information and might lead to an incomplete “picture” [11, 44]. Our observations for C19 patients having a better odour threshold in the second visit, a higher proportion of clinically relevant threshold improvement and, therefore, tended to have a higher TDI-score at the second visit might reflect the hypothesis that parosmia is caused due peripheral damage [11, 44] and could be a sign of regeneration [45]. Previous data showed the temporal relationship between regeneration and parosmia approximately 3–12 months after the onset of symptoms [14]. Moreover, the higher difference in threshold could underline the peripheral origin of parosmia and its activity at the level of the olfactory epithelium. In line with previous work [17], our data showed a trend of a better TDI-score in the second visit when C19 patients reported parosmia in at least one of the visits. Hence, our data showed for the first time that parosmia could be regarded as a positive prognostic predictor for a better odour threshold in the course of C19-associated OD. Another study showed for C19-associated OD examined in one visit that patients who developed parosmia at some point had a better olfactory function, as measured by the Brief Smell Identification Test approximately 6.5 months after the infection, but also a poorer quality of life [15]. For non-C19 patients, we did not find any relation of parosmia to the course of OD. For the non-C19 OD patient group, parosmia was less frequent overall and with a fewer number of patients in this group, so conclusions can only be drawn with limitations. The damage mechanism of more direct damage to olfactory receptor neurons in non-C19 might explain the difference in peripheral olfactory function.

Based on this hypothesis and on previous studies showing that parosmia may be related to regeneration, it would be interesting to relate parosmia also to the volume of the olfactory bulb [46]. It has been shown that the volume of the olfactory bulb is also associated with regeneration which could reflect central pathogenesis of parosmia. The present results underline the importance of the investigation of parosmia concerning the prognosis of OD, so that, among other things, the diagnosis of parosmia [47] should be established and investigated more intensively.

In the present data, predictors for an improving TDI were younger age (for C19), female gender (non-C19), and lower scores in olfactory tests (identification, discrimination, threshold, TDI) at the first visit. These findings are in line with previous studies, which showed that women had a better olfactory function than men [28, 48] and that there is an important correlation between olfactory function with age [28, 49]. In the group of C19 a better improvement of threshold was shown for younger patients, which could be explained by the known loss of olfactory neurons, replacement of olfactory neuroepithelium by respiratory epithelium, and a decrease in basal cell proliferation due to aging [50]. Specifically, the prevalence of olfactory dysfunction increases with age and can be up to 63% in 80–97 years [3, 51]. The results are, furthermore, in line with [19], who showed lower threshold, discrimination, identification, and TDI-score in the first visit for patients who had a clinically relevant improvement in the second visit. Patients with a higher TDI-score at the first visit may already have regained parts of their olfactory functions. At a second visit, their olfactory functions were then rather stable, without a significant impact on the final TDI score. Looking further at the predictors of normosmia, higher baseline TDI-scores were associated with a higher probability of normosmia [16] which is not reflected in the difference in the TDI-scores between both visits. This also implies that initially there would be a very fast improvement that becomes less obvious on a subjective, perceptual level and partly also on the level of clinical measurements of olfactory function.

We acknowledge that this multicentric study had some limitations. Improvements to be implemented in future studies would include: (1) the patient population for C19 and non-C19-associated olfactory disorders are different in terms of the absolute number of patients, comparative age, and duration of disease. This could partly explain the better olfactory function of C19 patients due to the above-mentioned association with olfactory function [52]. (2) Furthermore, the population bias should be mentioned. Only patients from outpatient clinics were included, who presented themselves because of a subjective severe olfactory loss. (3) In the present study, however, possible therapeutic regimens such as olfactory training have not been considered due to the different applications, and documentation in the multicentric design, so this remains unaccounted as a factor for the outcome. Olfactory training, for example, can improve the processing of olfactory information [53], functional connectivity within the olfactory system [54] and structural changes [55]. In this article, we focused on the course of olfactory dysfunction without a randomized or controlled paradigm for treatment, so that a spontaneous recovery rate is included.

## Conclusions

Patients with olfactory loss exhibit improvement over 3–4 months which seems to be similar between C19 and non-C19 URTI. A better prognosis in terms of olfactory recovery was found for C19, especially for younger patients who had parosmia and lower olfactory scores at the first visit. Parosmia in C19 patients seems to have a pronounced effect on the threshold in the course of OD which might be explained by the theory of peripheral damage for parosmia. Still, for a number of patients with olfactory loss, an improvement that is experienced as complete may only occur over months and possibly years.

## Data Availability

We will provide data upon request.

## Declarations

### Permission to reproduce material from other sources

N.A.

### Clinical trial registration

N.A.
